# A Practical Treatise on Acute Abdominal and Pelvic Inflammation

**Published:** 1830-04-01

**Authors:** 


**A PRACTICAL TREATISE ON ACUTE ABDOMINAL AND PELVIC INFLAMMATION; CONTAINING A COMPREHENSIVE CLINICAL VIEW OF INFLAMMATION OF THE STOMACH, BOWELS, PERITONEUM, UTERUS, &C. WITH A CERTAIN AND EXPEDITIOUS METHOD OF CURE. By *David Nicholas Bates*, Medical Practitioner. 8vo. pp. 136, London, 1829.**

THE words "certain and expeditious," applied to the cure of such dangerous phlogoses as those of the abdominal viscera, raised some doubts in our mind—but these doubts were quickly dissipated by a quotation from the LANCET, vouching for the efficacy of the plan proposed by Mr. Bates. Knowing, as we metropolitans do, the extensive practice, the experienced judgment, the accurate discrimination of our esteemed contemporary, Mr. Thomas Wakley—convinced that he would not hazard his well-earned reputation in the treatment of *inflammatory affections*, by vouching for any new practice before he had put it to the test of repeated experience, we took up the volume and read it through—partly in the regular vulgar way—partly in the way in which the great Napoleon declared he read the greater part of books—with his thumb. We shall now present our readers with all that is new—or thought to be new in this volume. To the classification which Mr. Bates has adopted we make no objection. The following passage is rather curious, and occurs at the very outset.

"Acute Inflammation, seldom in this country, attacks the *substance* of the Liver, Spleen, Pancreas, or Kidneys, at least, to any great extent, *as there is not sufficient sensibility in these parts to support it*; it seems to require all the susceptibility of the delicate mucous and serous membranes, to enable this disease to develop its peculiar characteristics." 3.

After detailing the symptoms of peritoneal inflammation, according to the site of the disease, which symptoms are taken from the common stock-books of the day, Mr. Bates notices the different terminations—the last of which, "and one of the most common," he tells us is—death.

"It appears to me, from the consideration of these circumstances, that death, in this disease, is not *immediately* occasioned by any appreciable organic lesion; as this has been found, after death, to have existed, in one or more of the viscera, without producing an effect upon the general system, equal to its extent, and even without being suspected.

"There must, however, exist in the system, more or less of *constitutional irritation*, in every departure from healthy action, whatever may be the degree, or nature, of such morbid action; whether it be a disease, attended with the most acute and agonizing pain, or one that occasions the most trifling degree of uneasiness.

"*Constitutional Irritation* is known to exist, by the disturbance, more or less, of the vital, natural, and animal functions; and, more particularly, in that set of organs, in which the disease resides. In this unnatural state of the system, there is always a corresponding *general debility*, proportionate to the importance of the part attacked, its sensibility, the continuance of the morbid action, and the degree of *constitutional irritation*, consequently existing.

"No morbid actions are attended with such remarkable constitutional irritation, as those which are productive of acute and continued *pain*; and none, therefore, more speedily bring the system into a state of extreme exhaustion. Even when natural actions are prolonged beyond the limits imposed by the laws of the animal economy, more particularly when accompanied with *pain, fatal exhaustion is the consequence*; for instance, the parturient female, who dies undelivered, when neither hæmorrhage, nor other untoward circumstances, has supervened, falls a victim to fatal exhaustion, *indirectly* induced by *pain*." 31.

The foregoing doctrine leads directly to the novel (as is supposed) practice of our author. The treatment may be laid before our readers in a very short compass. It consists in a moderate abstraction of blood, if the patient's strength will admit of it, and when the disease is early seen—the administration of opium, either by the mouth or by enema—the horizontal posture rigidly maintained—and thin diluents, with strict abstinence.

The following extract will explain the enema plan, which, upon the whole, our author seems to prefer.

"The *first plan*.

"The HORIZONTAL POSTURE must be strictly enjoined to the patient, and persisted in, while any symptoms of the disease remain; not the slightest departure from it must be permitted, even for the purposes of natural relief; it is, therefore, necessary for the patient to be provided with a bed-pan. The practitioner must always bear in mind, that this position is the "*sine quâ non*," without which, the remedies will be counteracted, and the cure of the complaint be frustrated, or protracted.

"The patient should lie on a soft bed, in order to encourage perspiration, as this is very favourable to the resolution of the disease.

"BLEEDING at the arm will be generally proper, when first called to a patient labouring under this disease, provided the strength will admit of it; but if there be great exhaustion, from the long continuance of the affection, from previous depletion, excessive vomiting, or purging, however the latter may be occasioned, the practitioner had then better refrain from blood-letting, and have immediate recourse to the opiate enema, which will next come under consideration. The quantity of blood taken, should not exceed a pint; and if the plan here generally recommended be adopted, it will not require a repetition. The buffy appearance of the blood, so often dwelt upon by authors, and considered by many medical men, as the grand test by which the danger of the attack can be estimated, ought not to influence the practitioner in the treatment of this disease: he must attend to the *violence of the pain*, together with the *vomiting*, or *purging*, if present, by which alone he will be enabled to form any thing like a just and certain prognostic; for it has often occurred to me, to see the blood exhibit no buffiness, when the symptoms left no doubt of the existence of most acute inflammation.

"The OPIATE ENEMA should be used immediately after the bleeding, or without it, if that evacuation be judged either unnecessary, or improper; the following is the form and quantity found most convenient and efficacious:

℞. Tr. Opii ʒj.—ʒij.*
Decoct. Amyli Calefact ℥xij.
Mf. Enema quàmprimum injiciendum.

"The general effect of this in quelling the *pain, sickness,* and *diarrhœa,* if the latter be present, is almost instantaneous; the patient who, previous to its exhibition, had been writhing under the agonies of acute inflammation in the stomach and intestines, or peritoneum generally, with continual retching and vomiting, and, perhaps, diarrhœa, at once becomes calm and composed, and complains only of great tenderness in the abdomen, more particularly in that part which was the seat of the disorder. If there be no return of pain in twelve hours, another enema, containing not less than a dram of tinctura opii, should be exhibited, to prevent a return; but if there be a renewal of the primary symptoms, at whatever period, the quantity first used may be again repeated. The tenderness of the body will be found to continue for a day or two, after the subsidence of the pain, but will gradually diminish, until it finally ceases. It will be necessary to confine the patient to the horizontal position, for not less than two days, after all the symptoms have disappeared." 37.

Directing cold water, or toast-water, as almost the only food as well as drink, Mr. Bates prescribes laxative lavements to relieve the bowels when confined—but not before the pain and sickness have been removed, and the tenderness of the abdomen somewhat abated. Leeches are also to be applied to the part "where the tenderness chiefly resides," while warm fomentations, and even anodyne stimulant liniments are to be put into requisition. We must make room for another extract descriptive of the "second plan."

"What has been said of Blood-letting, in the treatment just described, applies equally well in this.

"Instead of using the opiate enema, the following bolus is to be given:

℞. Pulv. Opii.

—— Antimonialis.

—— Acaciæ āā. gr. j.

Conf. Rosæ Caninæ q. s.

"Fiat bolus quàmprimum sumendus, in horâ repetendus, et binis horis continuandus, donec dolor cessat.

"If any one of the boluses be rejected by vomiting, another should be given immediately; as it is of consequence to ascertain whether the boluses be retained, the ejecta should be received into a white bason.

"By pursuing this plan steadily, it will be found, that the patient will gradually lose the pain and sickness, and, from the fifth bolus to the tenth, become tolerably easy; sometimes the third or fourth bolus will have the desired effect; but if, from any untoward circumstance, the tenth or twelfth bolus do not give decided relief, recourse must be had to the use of the opiate enema, as early as possible, as the longer the pain continues, the more likely is irreparable mischief to ensue." 43.

A number of cases illustrating the practice above-described, concludes the volume.

Mr. Bates, and the experienced editor of the Lancet do not seem to entertain the least question respecting the entire originality of this plan of treat-

---

* "This is the quantity for an adult; not less than Tr. Opii ʒj. should be used for the most delicate person of either sex: ʒiss. or ʒij. may be employed when the patient is of a very robust constitution, or the symptoms very violent. The lesser quantity has generally been found sufficient."

ing abdominal inflammations. Yet if they could spare an hour or two from their extensive practice to "search the journals," as parliament men would say, they might probably stumble upon some papers containing doctrines and practices not very unlike what have here made a volume. At present we would only direct their attention to Dr. Armstrong's paper on the Use of Opium in Inflammation, published in the first—and we fear the *last* volume of the "Transactions of the Associated Apothecaries," some seven years ago. One short extract will suffice.

"So great indeed is my confidence in full doses of opium in peritoneal enteritis, that if, compelled to say, supposing myself the subject of the disorder, whether I would exclusively rely upon them solely, or upon blood-letting solely, I should certainly fix upon the former; at the same time I should like to have the simultaneous influence of both remedies, being convinced, that they are far more serviceable combinedly, than separately employed." 315.

Mr. Bates indeed may attach great importance to some shades of difference between himself and Dr. Armstrong—such as the strict enjoinment of the horizontal posture—an injunction which common sense would enforce, without laying it down formally in print—the employment of opium in injections, as even preferable to an exhibition by the mouth—the *moderate* detraction of blood, &c.—but every candid man will say that these are mere modifications of the same principles and practice—and whether Mr. Bates' modifications are superior to those that have been recommended by Dr. Armstrong and others, can only, *as yet*, be pronounced by such luminaries as Mr. Wakley, whose talents and experience have long rendered him an ORACLE, on all practical points, among "medical practitioners" in this country.

The little work induced suspicions of something charlatanic about the performance; but we will do Mr. Bates the justice to say that we found nothing of the kind, on further examination. We have great doubts, however, as to the safety—and still more as to the efficacy of the practice advocated in the volume just reviewed. Of the dangers of abdominal inflammation we are as much aware as Mr. Bates can be—but we are not sure that this very limited depletion, (sixteen ounces of blood) and the masking of the symptoms by opium, may not sometimes throw practitioners off their guard till the disease has made a progress beyond the reach of medical art. After *copious* bleeding indeed, we have always advocated the administration of opium, particularly in combination with calomel—but this is a modification of great importance in the treatment of the disease under consideration, and the merits of the two plans must be left to time and future experience.

